# Imaging RAGE expression in atherosclerotic plaques in hyperlipidemic pigs

**DOI:** 10.1186/s13550-014-0026-6

**Published:** 2014-06-11

**Authors:** Lynne L Johnson, Yared Tekabe, Maria Kollaros, George Eng, Ketan Bhatia, Chong Li, Christian G Krueger, Dhanansayan Shanmuganayagam, Ann Marie Schmidt

**Affiliations:** 1 Department of Medicine, Columbia University Medical Center, 622 West 168 St, New York 10032, NY, USA; 2 Department of Medicine, NYU Medical Center, New York 10016, NY, USA; 3 Department of Veterinary Medicine, University of Wisconsin, Madison 53706, WI, USA

**Keywords:** RAGE, Atherosclerosis, Hyperlipidemic pigs, Imaging

## Abstract

**Background:**

Receptor for advanced glycated end product (RAGE) expression is a prominent feature of atherosclerosis. We have previously shown in apoE null mice uptake of a radiolabeled anti-RAGE antibody in atherosclerotic plaque and now evaluate RAGE-directed imaging to identify advanced plaques in a large animal model.

**Methods:**

Nine hyperlipidemic (HL) pigs were injected with 603.1 ± 129.5 MBq of ^99m^Tc-anti-RAGE F(ab′)_2_, and after 6 h (blood pool clearance), they underwent single-photon emission computed tomography/computed tomography (SPECT/CT) imaging of the neck, thorax, and hind limbs. Two HL pigs received ^99m^Tc non-immune IgG F(ab′)_2_, and three farm pigs were injected with ^99m^Tc-anti-RAGE F(ab′)_2_. After imaging, the pigs were euthanized. The aorta from the root to bifurcation was dissected, and the innominates, proximal carotids, and coronaries were dissected and counted, stained for H&E and RAGE, and AHA-classified.

**Results:**

On pathology, 24% of the arterial segments showed AHA class III or IV lesions, and these lesions were confined almost exclusively to coronaries and carotids with % stenosis from 15% to 65%. Scatter plots of %ID/g for class III/IV vs. I/II lesions showed almost complete separation. Focal vascular uptake of tracer visualized on SPECT scans corresponded to class III/IV lesions in the coronary and carotid vessels. In addition, uptake in the hind limbs was noted in the HL pigs and corresponded to RAGE staining of small arteries in the muscle sections. Correlations for the vascular lesions were *r* = 0.747, *P* = 0.001 for %ID vs. %ID/g and *r* = 0.83, *P* = 0.002 for %ID/g vs. % RAGE staining.

**Conclusions:**

Uptake of radiolabeled anti-RAGE antibody in coronary and carotid fibroatheroma and in the small arteries of the hind limbs in a relevant large animal model of atherosclerosis supports the important role of RAGE in atherosclerosis and peripheral artery disease as a target for imaging and treatment.

## Background

Receptor for advanced glycation end products (RAGE) is a multi-ligand receptor that plays an important role in the initiation and progression of atherosclerotic plaque and in mediating vascular inflammation in a variety of conditions [[1],[2]]. It is constitutively expressed in low levels on smooth muscle cells and endothelial cells in vascular endothelium, and the expression of RAGE increases in the vascular wall in response to a number of stimuli including hyperlipidemia and hyperglycemia [[3]].

Studies investigating RAGE expression in mouse models of atherosclerosis have used tissue extracts for protein analysis and quantitative immunohistochemistry [[4],[5]]. While these molecular biology tools are standard, they are limited in showing the extent and distribution of RAGE expression in the entire animal and require post-mortem tissue for analysis. Molecular imaging can show the distribution and intensity of the probe signal over the entire animal to identify the location and extent as well as the semi-quantitative expression of the target in an intact animal. This imaging approach is well suited to describe the expression of a receptor such as RAGE in atherosclerosis, a disease involving the entire arterial vascular tree.

We developed a radiolabeled monoclonal antibody fragment targeting a unique peptide sequence on the extracellular domain of the RAGE receptor and have shown in mouse models of atherosclerosis (apoE null) uptake on in vivo nuclear scans located in the sites of the atheroma in the aortic root and proximal aorta [[6],[7]]. In developing a new radiotracer for possible clinical use, it is necessary to show the results of in vivo imaging in a large animal model. Swine are commonly used in cardiovascular research due to similarities in the coronary anatomy and cardiac function to that of humans. Hyperlipidemic pigs bred in the domestic swine background have become a useful large animal model of atherosclerosis [[8]]. The purpose of this study was to perform RAGE-directed SPECT imaging to test the hypothesis that the signal from the uptake of the radiolabeled probe in atheroma can be detected in a large animal model of atherosclerosis comparable to humans and to investigate the extent and distribution of RAGE expression in the arterial vascular tree and localize to atheroma.

## Methods

### Animals

This study was designed as a single group of hyperlipidemic (HL) pigs for two purposes: to evaluate the extent and severity of atherosclerosis and RAGE expression in this large animal model and to see whether the signal from a radiolabeled antibody coming from this receptor can be visualized on in vivo imaging and correlated with quantitative histomorphometry. Four farm pigs were used as disease control. Specificity was determined in two HL pigs injected with isotype control antibody. All animal experiments were performed with the approval of the Institutional Animal Care and Use Committee of Columbia University. Nine juvenile male LDL-deficient (Rapacz) swine were sent to us from the University of Wisconsin-Madison. Seven arrived at age 3 months and were maintained in house for approximately 9 months or to 1 year of age, and two HL pigs arrived at age 11 months and were maintained in house for 1 month. Four age- and weight-matched farm pigs were also studied for blood pool clearance and as disease controls for imaging and ex vivo well counting.

All pigs received a high-fat swine diet (15% lard, 1.2% cholesterol) (Harlan Teklad, Madison, WI, USA). Weights were obtained monthly, and on the same day, the animals were sedated for venous blood samples for fasting glucose, blood chemistry profile, and lipid profile.

### Anti-RAGE antibody

The anti-RAGE antibody is a murine monoclonal antibody against the V-domain of RAGE designed to display immunoreactivity in mice, pigs, and human. The peptide sequence and production of the murine hybridoma has been described [[6]]. The monoclonal anti-RAGE antibody was fragmented using pepsin digestion into F(ab′)_2_ fragments (approximately 110 kDa) and immunoreactivity tested by ELISA using soluble RAGE antigen. Direct coupling of diethylenetriaminepentaacetic acid (DTPA) (bicyclic anhydride) to anti-RAGE F(ab′)_2_ antibody fragments for ^99m^Tc labeling was performed as previously described [[6],[7]]. The mean specific activity was 8.14 ± 3.8 MBq per microgram of protein, and the mean radiopurity was 98% ± 0.83% by instant thin-layer chromatography.

### Blood pool clearance

Ear vein catheters were placed in both ears of four farm pigs. Into one ear vein, an average dose of 16 mCi of ^99m^Tc-anti-RAGE F(ab′)_2_ was injected, and from the opposite ear vein, 1-ml samples were withdrawn at 2, 5, 10, 15, 20, 30, 45, 60, 90, 120, 180, 240, 300, and 360 min. From each tube, 50 μl was pipetted into pre-weighed tubes and counted in a gamma well counter. The counts vs. time were averaged for each time point and plotted. These pigs were also used for in vivo imaging and ex vivo well counting and histology.

### Radiotracer injection and imaging

At approximately 1 year of age, each HL pig was sedated, and angiocatheters were placed in both ear veins. A dose of 16.3 ± 3.5 mCi (0.3 ± 0.1 mCi/kg, 658.6 ± 148.0 MBq, 63.6 ± 13.3 MBq/kg) of ^99m^Tc-anti-RAGE F(ab′)_2_ antibody in 5-ml saline with a flush was injected through an ear vein. Two of these nine HL pigs also received ^99m^Tc non-immune IgG(Fab′)_2_ as well as ^99m^Tc-anti-RAGE F(ab′)_2_, 1-week apart with SPECT imaging following both injections, but the tissue was obtained after the control antibody. Four weight-matched farm pigs received injection of ^99m^Tc-anti-RAGE (17.5 mCi, 0.4 mCi/kg, 653.7 MBq, 12.9 MBq/kg) and were imaged.

### Imaging

After injection, the pigs were awakened and returned to their cages for 5-6 h to allow for blood pool clearance, then were re-sedated, intubated, and transported to the imaging laboratory where they first underwent CT angiography followed by SPECT/CT imaging (Philips Precedence 16 slice Hybrid SPECT/CT, Philips Healthcare, Andover, MA, USA). Each pig was injected with 100-ml non-ionic iodinated contrast agent Optiray 320 (Covidien, Mansfield, MA, USA) through the ear vein following test bolus. Scouts comprised neck to abdomen/pelvis. The following acquisition protocol was used: 120 kV, tube current 337 mA, collimation 16 × 0.75 mm, pitch 0.688, and slice thickness 0.8 mm. Acquisition trigger was set for 180 HU at aortic arch. Following CT angiogram, hybrid SPECT/CT imaging was performed with bed positions to include the chest, neck, and hind limbs. Each SPECT scan was set for two heads mounted at 180° for 64 steps over 360°. At completion of the imaging, the pigs were returned to the necropsy suite of the Institute of Comparative Medicine and euthanized with a bolus of Euthasol (100 to 120 mg/kg IV; Virbac Animal Health, St. Louis MO, USA).

### Scan analysis

The SPECT scans were reconstructed using filtered back projection, and the CT and SPECT images blended using Syntegra software (Philips, Andover, MA, USA). Focal regions of tracer uptake were localized to the territory of the carotids on sagittal and coronal blended SPECT/CT scans, and regions of interest were drawn around the uptake on the coronal slices to comprise the area of focal uptake excluding the most cephalad and caudad slices to reduce partial volume effect, and the counts from these regions were summed and converted to %ID using measured camera efficiency values and decay times. Because of the very low myocardial uptake, focal hotspots in coronary artery territories were visualized and localized on the blended chest CTA/SPECT images (Syntegra). For the hind limbs, the ROIs were drawn around sequential 1-voxel thick transverse slices using the blended SPECT/CT to define limb boundaries from the proximal femur to the distal tibia/fibula, and the activity from all the slices were summed for each limb.

### Necropsy and tissue preparation

The chest and abdomen were opened, the blood was drained from the vasculature, the heart was removed, and the coronaries from the opening of the coronary sinus to distal vessel were dissected out and rinsed with PBS. The aorta was dissected and removed from the aortic valve to beyond the iliac bifurcation to include the proximal femoral arteries and the major arch vessels (the brachiocephalic trunk (innominate artery) and carotid arteries). The tissue was cleaned and rinsed carefully and placed in a plastic tray kept moist in 10% formalin solution and placed on one of the detectors used for the in vivo scanning and imaged for 20 min. After imaging, representative cross-sections of the arteries were cut and labeled for well counting and sectioning. Each coronary was cut in four segments. The aorta was sampled as full-width cross-sectional segments from the aortic root through the abdominal aorta for a total of 12 segments per animal. The samples were taken from the proximal, mid, and distal innominate, from the proximal and mid right and left carotids, and from the proximal and mid femoral arteries. In addition, three samples were taken from each gastrocnemius muscle (six samples per pig). Each sample was weighed and counted in the gamma well counter (Wallac Wizard 1470, PerkinElmer, Waltham, MA, USA) along with an aliquot of the injected dose as standard and the %ID/g calculated for each sample and subsequently embedded in paraffin for sectioning. In selected animals, the lungs, liver, and heart were removed, weighed, and counted in the gamma well counter and then embedded in paraffin for sectioning.

### Immunohistology

For immunohistochemical analyses, serial sections were deparaffinized in xylene, treated with 0.3% hydrogen peroxide for 20 min, and incubated in protein-free block (Dako Inc., Carpinteria, CA, USA) for 10 min to inhibit the non-specific binding of primary antibody. All sections were stained with hematoxylin and eosin (H&E). Staining for RAGE was performed using monoclonal anti-RAGE antibody (50 μg/ml). Macrophages were identified using marker Mac-3 (1:20; BD Pharmingen, San Diego, CA, USA). Smooth muscle cells were identified using monoclonal mouse anti-human smooth muscle actin (1:50; Dako Inc.). Secondary staining was performed with HRP-conjugated respective secondary antibody, followed by diaminobenzidine (DAB substrate kit for peroxidase; Vector Laboratories, Burlingame, CA, USA), and counterstaining with Gill's hematoxylin solution.

Morphometric and immunohistochemical analyses of the arterial segments were performed using a Nikon microscope (Tokyo, Japan) and Image-Pro Plus software (Media Cybernetics Inc., Silver Spring, MD, USA). The lesion was measured as percent lesion area per total area of the aorta. RAGE staining was quantified as percent RAGE staining in the lesion area per total area of the aorta. Lesion morphology was classified according to the American Heart Association (AHA) criterion from class I to class IV [[9]].

### Statistical analysis

All data are presented as mean ± standard deviation. Correlation was assessed using the Pearson product–moment correlation.

## Results

### Weights and blood tests

For the nine HL pigs, the average arrival weight was 26.1 ± 4.8 kg, and the final weight was 63.6 ± 12.3 kg. The average cholesterol levels at arrival and sacrifice were, respectively, 460.4 ± 93.1 mg/dL and 445.6 ± 72.4 mg/dL, triglyceride levels 54.1 ± 15.3 mg/dL and 47.1 ± 12.1 mg/dL, and fasting blood glucose 71.0 ± 9.3 mg/dL and 88.4 ± 20.9 mg/dL. The weight of the farm pigs at time of imaging averaged to 50.7 kg. All lab values for the farm pigs were within normal limits for species.

### Blood pool clearance and biodistribution

**Figure 1:**
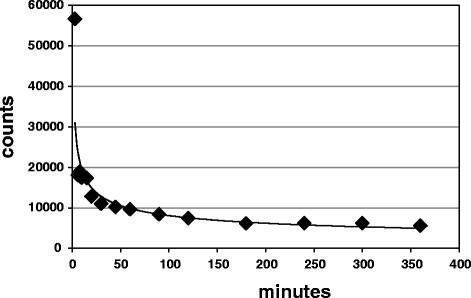
**Blood pool clearance of**^**99m**^**Tc-anti-RAGE F(ab′)**_**2**_**in a farm pig.**

The *t*_1/2_ for the first component was 13 min, and for the second component, it was 220 min (Figure 1). The lungs, heart, and liver were removed and weighed, and representative tissue samples were taken and counted in the well counter to determine the %ID/g of tissue. The lung activity was high without difference between the farm pigs and HL pigs representing high constitutive expression of RAGE in the lungs of domestic swine. Myocardial expression was low (averaging 0.03% of the ID). The liver uptake averaged at 16% ID.

### Location and extent of atherosclerotic lesions on histopathology

**Figure 2:**
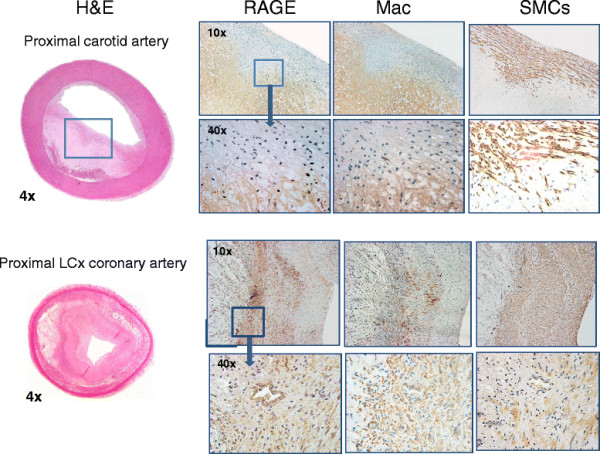
**Vascular pathology.** The top set of panels shows, from left to right, a cross-section of an H&E-stained carotid artery at ×4 magnification showing a thick cap fibroatheroma of proximal carotid artery and selected regions of the lesion in ×10 and ×40 with staining for RAGE, macrophages (Mac), and smooth muscle cells (SMCs). The lower panels show, on the left, the H&E sections from a proximal left circumflex lesion (LCx) (same lesion shown in Figure 3) and selected regions stained for RAGE, macrophages, and smooth muscle cells. The secondary antibody with HRP stained the target cells a reddish brown color.

For the 324 arterial segments sampled on the nine HL pigs, 283 were good tissue sections. On histology of these sections, 35 showed AHA class III or IV lesions (12%), 34 class II lesions (12%), and the remainder class I or no disease. Occlusive lesions were non-calcified fibroatheromas (Figure 2). The aortic root, arch, and thoracic aorta all showed AHA class I and II disease with only several class III lesions in the thoracic aorta. The class III and IV lesions were almost exclusively observed in nine coronaries, ten carotids, and six femoral arteries. The mean percentage stenosis for the coronaries was 39% ± 13% (range from 28% to 65%), for the carotids 42% ± 15% (range from 15% to 57%), and for the femoral arteries 36% ± 11% (range from 20 to 51).

### Uptake of ^99m^Tc-anti-RAGE F(ab′)_2_ in carotid and coronary arteries

**Figure 3:**
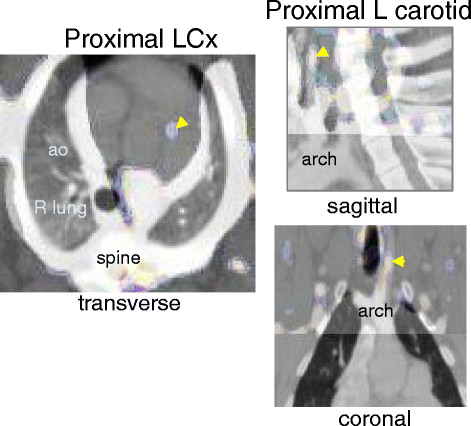
**SPECT/CT images.** The left image is a transverse blended image at the level of the aortic root showing focal uptake corresponding to the origin of the LCx. The middle and right images show the sagittal and coronal blended images at the level of the anterior neck showing focal uptake that localizes to the left carotid artery, respectively. Both areas of focal uptake corresponded to higher values for %ID/g from well counter and IV AHA lesions on histology.

Focal uptake of tracer was noted in regions corresponding to nine coronary arteries and ten carotid vessels. The calculated %ID for the coronaries was 1.2 ± 0.4, and for the carotids 0.7 ± 0.3. All of these vessels, when excised and sectioned, showed AHA class III and IV atherosclerotic lesions. Examples of scans showing focal uptake in the proximal left circumflex (LCx) and left (L) carotid are shown in Figure 3.

**Figure 4:**
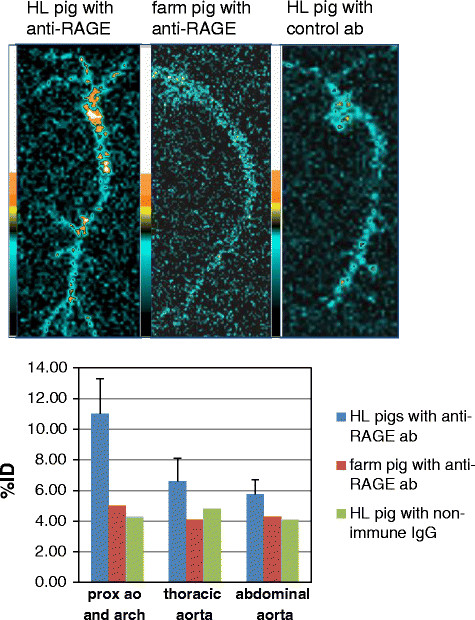
**Ex vivo imaging of the aorta.** The top-row images are the planar scans of the aortas from a HL pig injected with ^99m^Tc-anti-RAGE F(ab′)_2_ (left), farm pig injected with the same antibody in the middle, and a HL pig injected with radiolabeled control antibody on the right. Tracer uptake is seen in the distal arch and proximal thoracic aorta in the HL pig, but no appreciable focal uptake is seen on the aortas from the farm pig and the HL pig injected with control antibody. The graph below shows uptake as %ID, with blue bar representing the mean ± SD for all the HL pigs injected with ^99m^Tc-anti-RAGE F(ab′)_2_ and average values for three farm pigs and two HL injected with non-immune IgG. The small increment of uptake of ^99m^Tc-anti-RAGE F(ab′)_2_ over the controls corresponded with low expression on histology.

Planar images of the aorta imaged on the detector are shown in Figure 4. The farm pigs injected with ^99m^Tc-anti-RAGE F(ab′)_2_ and HL pigs injected with ^99m^Tc-labeled control antibody did not show appreciable focal tracer uptake. The HL pigs showed areas of uptake localized to the distal arch and thoracic aorta corresponding to AHA II lesions on histology. The %ID calculated for segments of the excised aorta counted on the detector of the gamma camera for the proximal, mid, and distal aorta were 11.0% ± 2.3%, 6.6% ± 1.5%, and 5.75% ± 0.96% for the HL pigs, and for the disease and antibody control experiments were between 4% and 5% (Figure 4).

### Gamma well counting of vascular tissue

**Figure 5:**
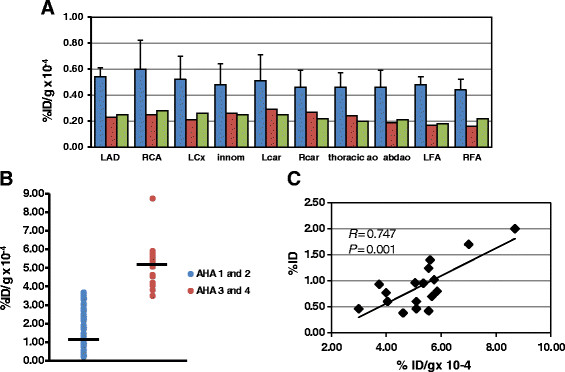
**Quantitative tissue uptake of radiotracer. (A)** Bar graph of distribution of uptake as %ID/g from the gamma well counting of vascular tissue from the HL pigs (blue bars) (mean ± SD) and averages of the controls. Innom, innominate artery; abd, abdominal; LFA, left femoral artery; RFA, right femoral artery. **(B,C)** Correlations between %ID from scan and % RAGE staining with %ID/g.

The 36 arterial sections from each pig were counted in the gamma well counter, and counts as %ID/g were calculated for all samples for the nine HL pigs, the four farm pigs, and the two HL pigs injected with non-immune IgG F(ab′)_2_. The average ± SD for vascular tissue sections with highest values for %ID/g for each vascular segment for the nine pigs are shown in Figure 5A. The distribution of probe uptake corresponded to the distribution of atherosclerotic lesions with lowest uptake in the aortic root and arch, and the highest values for the coronary arteries and carotids.

To better assess the relationship between tracer uptake and AHA class, all histological sections from the innominate, left and right carotid, LAD, RCA, LCx, aortic root, arch, and thoracic aorta for each animal were grouped into class I and II lesions (minimal disease) and class III and IV lesions (more advanced). The tracer uptake as %ID/g from well counting for these sections were plotted as scattergrams (Figure 5B). There was minimal overlap of values for the two groups. Only two vessels with minimal disease had an uptake of >3.5% ID/g × 10^−4^, and only one with advanced disease had an uptake of <3.5 (Figure 5B). The uptake in all class III and IV lesions in the coronary and carotid vessels was visible on the SPECT scans, and none of these vessels with only class I and II lesions showed focal tracer uptake.

Values for %ID for the visible focal vascular uptake of ^99m^Tc-anti-RAGE F(ab′)_2_ were plotted vs. values for %ID/g for the same vascular segments counted on the gamma well counter. There was a significant correlation: *r* = 0.747, *P* < 0.001 (Figure 5C).

### Quantitative staining of atheroma for RAGE

Quantitative immunostaining for RAGE as % vessel area ranged from 5% to 20% and localized to media and neointima. Serial sections stained for endothelial cells, macrophages, and smooth muscle cells showed predominant co-localization of RAGE staining with smooth muscle cells and macrophages (Figure 2). When % RAGE staining was plotted vs. vascular uptake of ^99m^Tc-anti-RAGE F(ab′)_2_, there was a significant correlation: *r* = 0.824, *P* < 0.001.

### Hind-limb uptake of anti-RAGE antibody

**Figure 6:**
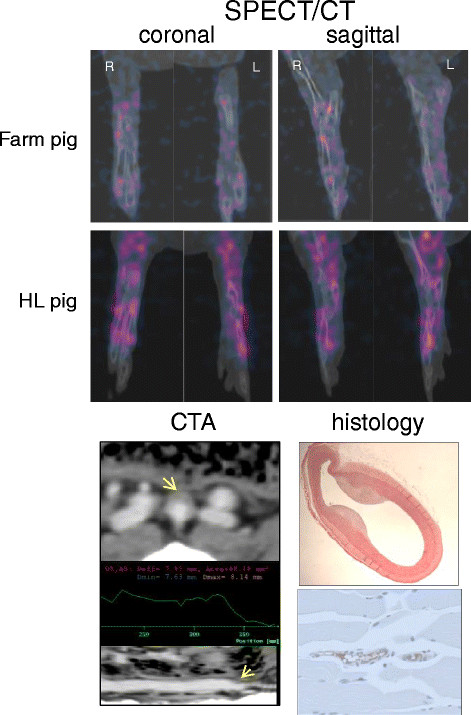
**Hind limb imaging and histology.** Top 4 images are coronal (left) and sagittal (right) slices from SPECT/CT scans of hind limbs of farm pig (above) and HL pig (below) injected with ^99m^Tc anti-RAGE F(ab′)_2_. There is diffuse tracer uptake in both hind limbs of the HL pig compared to low uptake in the farm pig. Bottom images on the left are the CTA showing atheroma at the iliac bifurcation in a HL pig and on the right the H&E-stained cross-sections from the femoral artery of a HL pig showing fibrous atheroma and from the gastrocnemius muscle of a HL pig showing RAGE staining in the small arterial walls.

All nine HL pigs showed non-obstructive fibroatheromas at the bifurcation of the distal aorta into the ileofemoral branches seen on histology (Figure 6), but visualizing focal uptake of the radiolabeled probe in these lesions was difficult due to the proximity to the bladder and bowel. In distinction to focal uptake seen in atheroma in large vessels, a pattern of diffuse tracer uptake was observed in the hind limbs of all the HL pigs. Uptake as %ID calculated as the sum of sequential 1-voxel-thick transverse slices from proximal femur to distal tibia/fibula for the nine HL pigs was 6.73 ± 0.80, and for the farm pigs, the average value was 3.75. The blended SPECT/CT coronal and sagittal images from one farm pig and one HL pig are shown in Figure 6. The gastrocnemius muscle immunohistology showed positive RAGE staining in the endothelial layer of small arteries on the biopsy specimens, and therefore, the diffuse pattern of tracer uptake on the scans is interpreted as RAGE expression in small arterial walls.

## Discussion

This paper is the first to report the potential value of imaging RAGE expression in atheroma throughout the vascular tree in a large animal model genetically altered to develop advanced atherosclerotic lesions similar in morphology to human disease [[8],[10]-[13]]. Using SPECT imaging, the focal uptake of a ^99m^Tc-labeled probe binding RAGE was seen in the distribution of AHA class III and IV lesions in the carotid and coronary arteries. No focal uptake was seen in regions corresponding to class I and II lesions. These findings were confirmed by ex vivo well counting of the vascular tissue. In addition, diffuse uptake of RAGE targeting probe was seen in the lower extremities and histopathology showed increase RAGE expression in small- and medium-size arteries.

RAGE is a multi-ligand receptor that binds non-enzymatically glycosylated proteins or AGEs and other ligands initiating downstream pathways important in atherogenesis [[14]-[18]]. Knocking out RAGE in atherosclerotic-prone mice reduces the development of atherosclerosis [[19],[20]], further supporting the importance of RAGE-initiated pathways in atherogenesis and plaque progression. Pathological studies of human autopsy or biopsy specimens have documented the importance of RAGE in atherosclerosis and peripheral artery disease. To localize and quantify RAGE expression, Burke et al. found RAGE expression in fibroatheromas from subjects with sudden cardiac death [[21]], and Cipollone et al. found RAGE expression in carotid plaque from patients with transient ischemic events [[22]]. A pathology study reported by Ritthaler et al. in 1995 documented prominent enhancement of endothelial RAGE expression in small-and medium-size arteries in the lower extremities of patients with occlusive peripheral vascular disease [[23]].

The development of both SPECT and PET probes targeting atherosclerosis towards the goal of finding a non-invasive screening test for subjects at highest risk for CV events (both myocardial infarction and stroke) has been a major focus for investigators in CV molecular imaging. As progress in molecular biology has identified an increasing number of potential sites for binding ligands, a large number of probes using nuclear, optical, ultrasound, and MR reporters have been developed for preclinical small animal studies [[24]]. Nuclear probes offer the advantage of small-size molecules that can gain access to the center of the lesion to bind to sites not accessible to larger probes that are confined to binding sites expressed on the vascular lining. The spatial resolution for the newer SPECT detectors even at the depth of the heart is at least twice the diameter of a coronary artery and PET resolution is at the border of the coronary diameter size. Despite limited resolution, focal uptake of tracers can be seen in small structures on in vivo scans as ‘beacons.’ The strength of the signal and ability to see it depend on the number of binding sites for the probe as well as attenuation and scatter from adjacent activity. Correlation with quantitative immunohistology allows us to identify a threshold level of target expression to permit in vivo imaging. The very fact that one can see this target indicates a high level of biological expression which would have clinical significance.

Hybrid imaging (SPECT/CT or PET/CT) when combined with angiography can optimize localization of focal tracer uptake to a vascular structure. Vascular uptake of ^18^ F-FDG tracks metabolic activity of plaque macrophages and therefore plaque inflammation, a pathological feature of vulnerability [[25]]. Because the myocardium relies on glucose as a substrate for metabolism, FDG has limited application for coronary imaging due to high background [[26]]. Carotid plaques are an easier target than coronary plaques due to larger size of vessels, less motion, and less attenuation. While focal uptake of FDG seen on PET/CT scans has been reported to correlate with plaque macrophages, limitations to ^18^ F-FDG carotid plaque imaging occur due to residual blood pool activity, adjacent tissue uptake, and effects of blood glucose levels on uptake [[27]]. While RAGE plays a role in a number of signaling pathways, its role in atherosclerosis is mediated mostly via inflammation pathways and, in this respect, similar to FDG. In contrast to FDG, ^99m^Tc anti-RAGE F(ab′)_2_ probe has very low myocardial uptake due to low constitutive RAGE expression in the non-ischemic myocardium. Based on our experience with RAGE lung tissue staining in different species including human, the high lung uptake observed which interfered with imaging the aorta, is unique to the pig. The wide availability of hybrid imaging platforms as well as software for accurate registration of SPECT or PET scans with CT scans now makes it practical to apply hybrid nuclear/CT atherosclerotic plaque imaging as selective screening for very high risk patients.

## Conclusions

The results of the current study documented in a large animal model of atherosclerosis with plaque characteristics similar to those of man that the focal uptake of a radiolabeled antibody targeting RAGE which plays an important role in atherogenesis and plaque vulnerability can be detected on in vivo imaging with a threshold for detection at AHA III and IV lesion severity. In addition, semi-quantitative uptake of tracer on in-vivo imaging localized to distribution of diseased coronary and carotid vessels correlated with gamma counting of the tissue and with quantitative staining for RAGE. Diffuse uptake of the radiolabeled tracer was seen in the hind limbs corresponding to RAGE expression in the small arteries. Because RAGE is a multi-ligand receptor and transduces many important signaling pathways in vascular disease and atherogenesis, it represents a potentially good target for imaging atherosclerosis and PAD.

### Limitations

The hybrid imaging would have been strengthened if we were able to perform coronary CT angiograms with gating to register with the SPECT images. We were unable to get the heart rates of the pigs low enough to permit good quality studies on a 16-slice CT scanner. While the whole body CTA we used to register with the SPECT to localize tracer uptake was ungated, the base of the heart where the origins of coronary arteries are located (Figure 3) has less cardiac motion than towards the apex, and any motion would serve only to further blur the focal hotspot but not reduce our sensitivity to detect it.

## Abbreviations

CTA: CT angiogram: 

DTPA: diethylene triamine pentaacetic acid: 

HL: hyperlipidemic: 

HMGB1: high mobility group box 1: 

ID: injected dose: 

LAD: left anterior descending: 

LCx: left circumflex: 

RAGE: receptor for advanced glycation end products: 

RCA: right coronary artery: 

ROI: regions of interest: 

SPECT/CT: single-photon emission computed tomography/computed tomography: 

## Competing interests

The authors declare that they have no competing interests.

## Authors’ contributions

LJ supervised the performance of the experiments and wrote the manuscript. YT prepared the radiotracer and did the well counting and histological staining. MK harvested the tissue and prepared it for histology. GE performed the necropsies. KB imaged the pigs. CL performed the quantitative immunohistology. CK and AS are responsible for the HL pigs at the University of Wisconsin. AMS is an expert in the field of RAGE biology and collaborated on the development of the imaging antibody. All authors read and approved the final manuscript.
